# Resection of brain radionecrosis after stereotactic radiosurgery or radiotherapy: a meta-analysis

**DOI:** 10.1007/s10143-026-04208-x

**Published:** 2026-03-14

**Authors:** Karun Donthineni, Hyejoong M. Lee, Prabodh Sankhe, Alice van den Broek, Semah Misconi, Charissa Jessurun, Marco Mammi, Marike L.D Broekman, Rania A. Mekary

**Affiliations:** 1 School of Pharmacy, Massachusetts College of Pharmacy and Health Sciences University, Boston, MA 02215 USA; 2 Department of Neurosurgery, Haaglanden Medical Center, Leiden University Medical Center, Leiden, The Netherlands; 3 Department of Radiotherapy, Amsterdam University Medical Center, Amsterdam, The Netherlands; 4 Department of Neurosurgery, “M. Bufalini” Hospital, Cesena, Italy; 5 Epidemiology Research Faculty, Computational Neuroscience Outcomes Center (CNOC), Harvard Medical School, Mass General Brigham, Boston, MA USA; 6 Department of Biostatistics, Harvard TH Chan School of Public Health, MA 179 Longwood Avenue, Boston, MA 02115 USA

**Keywords:** Brain radionecrosis, Radiation necrosis, Radiotherapy, Stereotactic radiosurgery, Surgical resection, Meta-analysis, High-grade glioma, Brain metastasis, Neurological outcome, Survival outcomes

## Abstract

**Background:**

Brain radionecrosis, a late-stage adverse effect of radiotherapy, presents diagnostic and treatment challenges. Although reports on its surgical resection have increased, no systematic review has thoroughly evaluated its clinical outcomes.

**Objective:**

To assess post-operative neurological improvement, overall survival, and complications following resection of brain radionecrosis.

**Methods:**

A search of PubMed, Embase, and the Cochrane Library was conducted following PRISMA principles (inception–February 2025). DerSimonian and Laird random-effects models were used to estimate pooled incidence with 95% confidence intervals (CIs).

**Results:**

Eight retrospective case series involving 443 patients, predominantly with high-grade gliomas and brain metastases, met the inclusion criteria. Neurological improvement was observed in 80.7% of patients (95%CI, 66.8%–89.6%). Survival outcomes were reported heterogeneously across studies. Only two studies provided overall survival (OS) from the time of resection in histopathology-confirmed pure radionecrosis cohorts; in these, pooled 12-month and 24-month OS were 84.6% (95%CI, 65.4%–94.1%) and 73.1% (95%CI, 53.3%–86.6%), respectively. The pooled incidence of postoperative complications was 21.4% (95%CI, 11.7%–35.9%), with most complications being transient and non-life-threatening, indicating an overall favorable safety profile.

**Conclusion:**

Surgical resection for brain radionecrosis appeared to provide meaningful clinical benefit, with high rates of neurological improvement and an acceptable incidence of postoperative complications. Although survival outcomes were reported inconsistently and with varying time origins, available data suggest that selected patients may experience favorable short-term survival following surgery. Standardized definitions of radionecrosis and uniform reporting of survival from the time of resection are needed to strengthen the evidence base and guide clinical decision-making.

**Supplementary Information:**

The online version contains supplementary material available at 10.1007/s10143-026-04208-x.

## Introduction

Radionecrosis of the brain (RN) is a well-documented late complication of stereotactic radiosurgery (SRS) and fractionated radiotherapy (RT), which are critical modalities for treating both primary and metastatic brain tumors [1]. The onset of RN can occur months to years after treatment, with an incidence that varies significantly, ranging from 3% to 24% following SRS [2]. RN is thought to be caused by radiation-induced vascular endothelial damage, disruption of the blood-brain barrier, and subsequent necrotic changes in irradiated brain tissue [3]. Clinically, RN often resembles tumor recurrence, presenting as seizures, mass effect, or new or worsening neurological impairments, necessitating advanced imaging or histological confirmation for diagnosis [4, 5].

RN is currently managed with conservative measures, including corticosteroids and anti-angiogenic drugs (such as bevacizumab), as well as surgical resection [6]. Other approaches, such as hyperbaric oxygen therapy or laser interstitial thermal therapy (LITT), have also been explored [7]. Medical treatments aim to reduce edema and inflammation, but their effectiveness is often limited, and long-term reliance on steroids carries serious side effects, including immunosuppression, myopathy, psychiatric disturbance, and metabolic complications [8]. Although surgical intervention is invasive, it provides immediate symptom relief by alleviating mass effect and offers a definitive histopathological diagnosis; however, it also carries risks of postoperative complications, such as infections and neurological deficits [9, 10].

Despite growing interest in the surgical resection of brain RN, much of the available research consists of retrospective case series suggesting neurological benefit and acceptable safety [7, 11, 12]. To clarify how outcomes vary by pathology (e.g., brain metastasis [BM], high-grade glioma [HGG]), we conducted a systematic review and meta-analysis of surgical outcomes for symptomatic RN, synthesizing evidence on neurological benefit, survival, and complication rates, and exploring how these outcomes differ by underlying tumor type.

This study aimed to systematically evaluate (1) postoperative neurological improvement, (2) overall survival, and (3) surgical complication rates in patients who underwent resection for cerebral RN. While we sought to explore how outcomes varied by pathology (e.g., brain metastasis [BM], high-grade glioma [HGG]) where data permitted, we recognized that heterogeneity in reporting and patient populations might limit fully stratified analyses. By synthesizing existing data, we aimed to provide the most comprehensive summary of surgical outcomes for RN to date and to identify critical gaps in the literature that should guide future prospective research.

## Methods

### Search strategy

This systematic review and meta-analysis were conducted following the PRISMA 2020 checklist. The MEDLINE (PubMed), Embase (Ovid), and Cochrane Library databases were used to conduct a thorough literature search. Medical Subject Headings (MeSH), along with relevant text words associated with “stereotactic radiosurgery,” “radiotherapy,” “radionecrosis,” and “surgical resection,” were included in the search strategy. Searches were conducted from inception to February 2025. **Appendix 1** contains a detailed explanation of the search terms and approach.

### Study selection

Studies were considered eligible if they: (1) involved human patients diagnosed with brain RN after receiving SRS or fractionated RT, for which they underwent neurosurgical resection, with histological confirmation of RN; (2) included a minimum of five patients; and (3) reported outcomes after surgical resection of radionecrotic tissue. Studies were excluded if they were conducted on animals, centered solely on pharmacological treatments, lacked full-text articles, or were published in a language other than English. Title and abstract screenings were conducted in Covidence (Veritas Health Innovation Ltd) independently by four reviewers (KD, HL, AB, SM) [13]. Full texts were screened by five reviewers (KD, HL, AB, SM, PS). Any discrepancies or conflicts during the screening process were resolved through consultation with a senior reviewer (MM, RM, MB).

### Data extraction

Data were extracted independently by two reviewers using a standardized Excel sheet (Microsoft Corporation, Version 16.0, 2024) (KD, HL). Extracted variables included study characteristics (author, year, country), patient demographics, primary tumor type (BM, HGG, or others), indication for surgery, imaging modality used for diagnosis, surgical technique, histological diagnosis (pure RN vs. mixed RN/tumor), and outcome measures. Clinical outcomes of interest included neurological status postoperatively (e.g., Karnofsky Performance Status [KPS]), radiographic response, survival metrics (e.g., overall survival), and postoperative complications. For time-to-event outcomes such as OS, if only Kaplan–Meier curves were provided, we digitized the curves using WebPlotDigitizer (version 5.2; [https://automeris.io/WebPlotDigitizer]) to estimate survival rates at specified time points [14]. This approach allowed us to estimate OS at clinically relevant time points for inclusion in the descriptive synthesis.

### Data synthesis and statistical analysis

Because all the studies included were single-arm retrospective case series, the analysis concentrated on determining pooled proportions (incidence) of critical outcomes linked to surgical resection for brain RN. Furthermore, the 95% confidence interval (CI) was calculated in the meta-analysis utilizing the DerSimonian and Laird random-effects model [15, 16]. Forest plots were created to visually depict the pooled incidence estimates. Heterogeneity was evaluated with the I² index, where values exceeding 40% suggested moderate to substantial heterogeneity [17, 18]. We conducted subgroup analyses by primary tumor type (BM, HGG, and mixed cohorts). When a subgroup contained one study, we reported that estimate descriptively without pooling. Statistical analyses were conducted using Comprehensive Meta-Analysis version 4 (Biostat, Inc) [19].

### Risk of bias assessment

The methodological quality and risk of bias of the included case series were assessed using the Joanna Briggs Institute (JBI) Critical Appraisal Checklist for Case Series [20]. This checklist examines the quality of the study across multiple areas, including demographic reporting, outcome measurement, inclusion criteria, and follow-up. Two reviewers (KD, HL) independently evaluated each paper, discussing and resolving any disagreements. With fewer than 10 studies included, the small study effect was not assessed using funnel plots or statistical tests such as Begg’s [21].

## Results

### Study selection

**Fig. 1:**
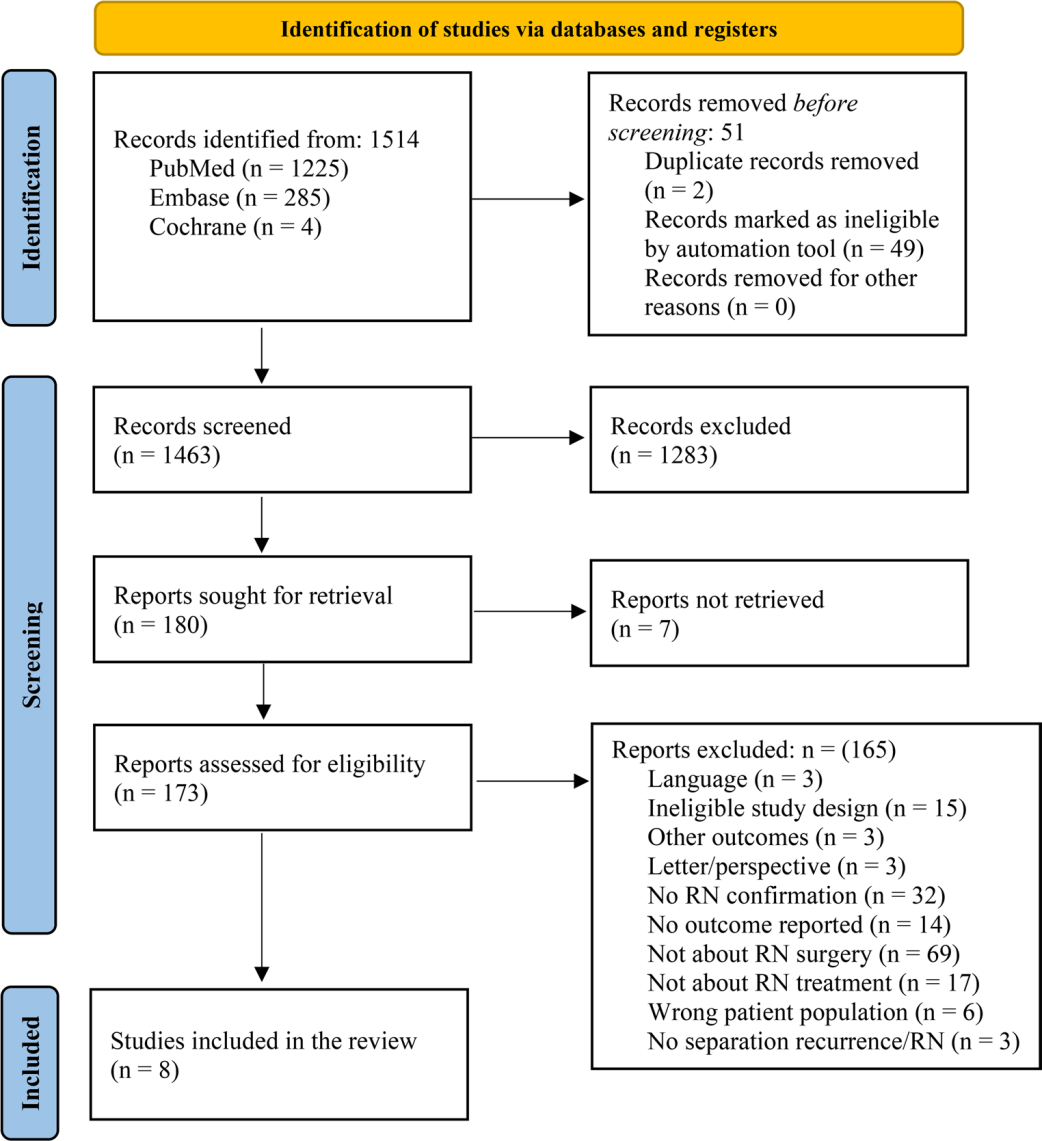
Prisma flow diagram for study selection

A total of 1,514 records were identified through database searches. After removing 51 duplicates, 49 automatically through Covidence and two manually, 1,463 unique records were screened. Among these, 1,283 studies were excluded for not meeting the inclusion criteria. Of the remaining 180 records, seven were conference abstracts without accompanying full-text publications. As no full articles accompanying the abstracts were available and the abstracts lacked sufficient detail for data extraction, these were excluded. Full texts were retrieved and reviewed for the remaining 173 articles. Detailed reasons for full-text exclusion were documented in the PRISMA flow diagram **(**Fig. 1**)** [22]. After a rigorous evaluation, eight studies met all eligibility criteria and were included in the final analysis [23–30].

### Characteristics of included studies

The eight included studies were retrospective case series, published between 2004 and 2024, and analyzed brain RN outcomes after brain tumor irradiation. The studies originated from various countries, including Italy [25], Israel [26], Germany [28], South Korea [29], and the USA [23, 24, 27, 30]; sample sizes ranged from 11 to 159 patients. Of the 443 patients who underwent surgery, 141 were histologically confirmed to have RN (Table 1).

Patient demographics indicated an average age of 49–60 years. The proportion of male patients ranged from 33% to 69% across series. Patients had various primary intracranial tumors needing radiation; about half were HGG, while others were treated for lung and breast cancer metastases. Importantly, the histological composition of RN pathology varied across studies. Three studies reported pure RN confirmed at resection, with pure RN sample sizes ranging from 7 to 27 patients [25, 29, 30]. Three studies included mixed pathology in which RN coexisted with a viable tumor or could not be clearly distinguished [23, 27, 28]. The remaining two studies focused primarily on HGG-associated RN, although definitions differed; Rusthoven et al., for example, used a ≥ 80% necrosis threshold, which does not strictly represent pure RN [24, 26]. This distinction is clinically relevant because pure and mixed RN are expected to differ in biological behavior, diagnostic certainty, and survival. Hence, the pathology subtype (pure vs. mixed RN) is reported in Table 1.

**Table 1:** General characteristics of the included studies

Study, Year; Country	Sample size	Age (mean| median; range | SD)	% male	Primary tumor type(s)	Previous radiation course	Follow-up	Overall Survival (as reported)
McPherson C, 2004; USA	11	Mean 50 (range 27–63)	64	Glioma (WHO III–IV); brain metastases	Fractionated RT 60 Gy; WBRT; SRS; brachytherapy	Mean 27 mo from initial RT (range: 7-108)	Median OS 13 mo (time origin not clearly specified; cohort of surgically treated RN)
Rusthoven K, 2011; USA	51 (14 pure RN)	Median 49 (range 22–71)	69	Glioma (WHO III–IV)	Fractionated RT (60 Gy) ± concurrent chemotherapy	Median 6.7 mo post-RT (range: 1–59)	Median OS 21.8 mo from reoperation (≥ 80% necrosis; not pure RN)
Telera S, 2013; Italy	15 (7 pure RN)	Mean 58 (range 35–72)	33	Brain metastases (lung, breast, renal)	Gamma Knife SRS ± WBRT	Median 14 mo (range: 6–38)	Median OS 19 mo (95% CI 7–49); pure RN: 12-mo 85.7%, 24-mo 68.6%
Grossman R, 2016; Israel	159 (18 RN group)	Mean ± SD 57.2 ± 9.7	62	Glioblastoma	Fractionated RT 60 Gy	NR	Median OS 15 mo from diagnosis (RN group)
Shah A, 2019; USA	24	Mean ± SD 60.3 ± 10.0	42	Metastases; high-grade gliomas; meningiomas; esthesioneuroblastoma	WBRT; fractionated RT; SRS	Mean 13.3 mo§	12-mo OS 93.3%; 24-mo OS 66.7%; median OS NR
Campos B, 2020; Germany	21	Mean 57 (range 42–72)	43	Metastases; meningiomas; gliomas; sarcoma/carcinoma of nasopharynx; others	SRS / fractionated RT	≥ 24 mo§	Median OS NR (focus on timing of necrosis and surgical safety)
Kim J, 2022; South Korea	86 (19 pure RN)	Mean 57 (range 40–77)	36	Brain metastases (lung, breast, renal)	Gamma Knife SRS	Median 27.7 mo§	Overall median OS 21.7 mo; pure RN: 12-mo 82%, 24-mo 75%
Bhatia R, 2024; USA	76 pts, 82 lesions (27 pure RN)	Median 56 at first SRS§	66	Metastases (NSCLC, breast, melanoma, renal, endometrial, thyroid)	SRS ± WBRT	Median 35.3 mo (range: 2.0–99.7)	Median OS 35.3 mo from first SRS (not surgery)

*Abbreviations*: *mo* months, *RT* radiotherapy, *WBRT* whole-brain radiotherapy, *SRS* stereotactic radiosurgery, *RN* radiation necrosis, *NR* not reported, *NSCLC* non-small cell lung cancer

§ Exact range not stated

All patients received high-dose brain radiation before developing RN. Glioma patients typically received fractionated radiotherapy (approximately 60 Gy in 30 fractions), while those with metastases often received SRS (e.g., Gamma Knife). The median time from radiation to RN diagnosis ranged from 6.7 to 35 months. Follow-up varied from 12 to 14 months to 2–3 years, with Bhatia et al. (2024) [30] citing 35 months. Despite variability, all studies discussed management challenges for patients with RN imaging and clinical signs, leading to surgical considerations.

### Neurological outcomes

**Fig. 2:**
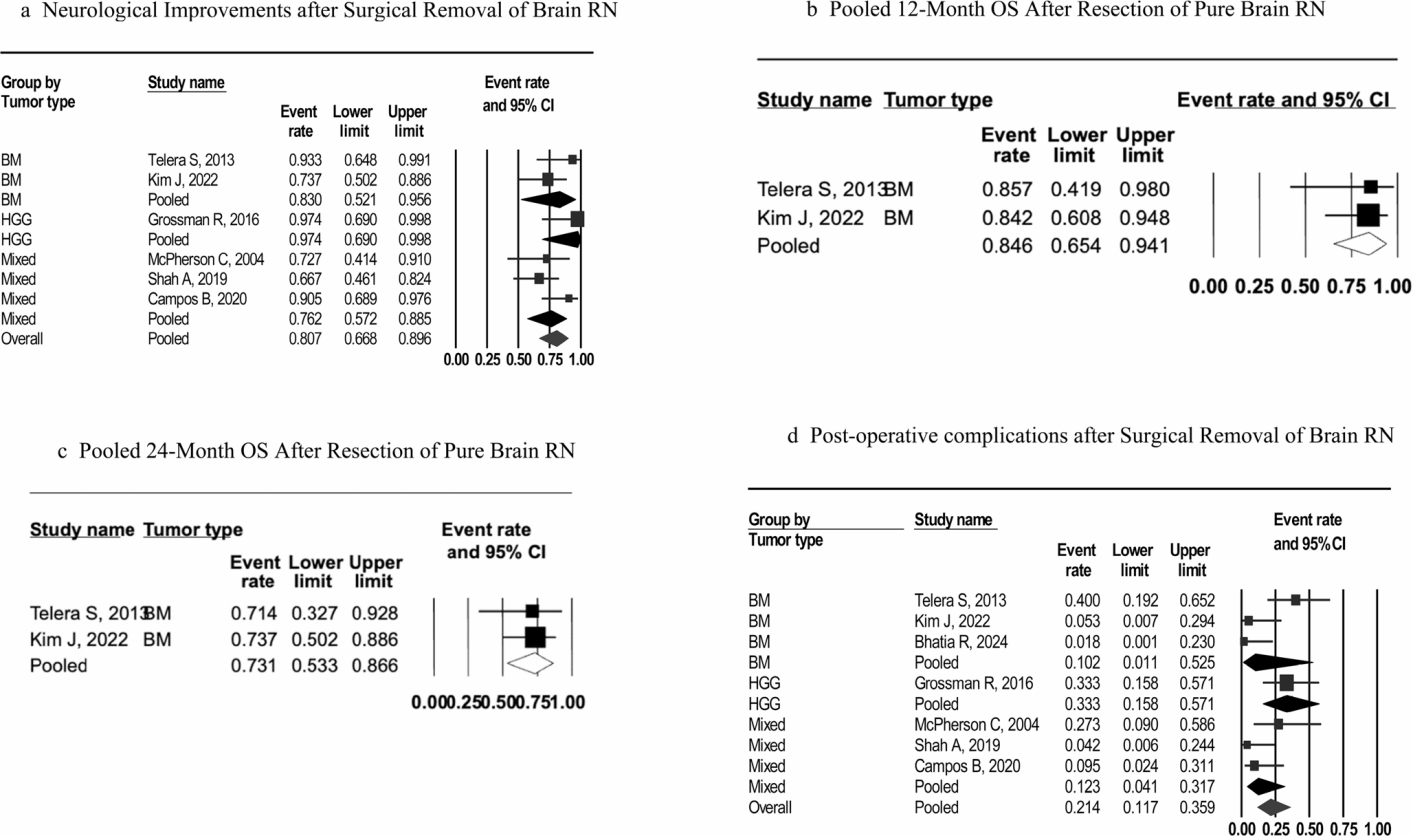
Pooled point estimates after surgical removal of brain RN. Figure 2**a** illustrates neurological improvement; Fig. 2**b** and **c** illustrate overall survival rates of pure RN cohorts at 12 and 24 months, respectively; Fig. 2dd illustrates postoperative complications, including motor and speech and language difficulties, cognitive impairments, seizures, and visuospatial deficits. All forest plots depict pooled estimates using the DerSimonian and Laird random-effects model. Squares represent individual study estimates with size proportional to study weight; horizontal lines indicate 95% CIs. Diamonds indicate the pooled estimates across studies and overall. Abbreviations: BM, brain metastasis; CI, confidence interval; HGG, high-grade glioma; OS, overall survival; RN, radionecrosis.

All studies examined neurological outcomes related to managing RN, particularly symptom improvements post-surgery. In the included series, most patients showed postoperative neurological improvement (Appendix 2). Neurological improvement was variably defined across studies, including higher KPS scores, resolution of focal neurological deficits (e.g., motor or visuospatial function), reduced seizure frequency, or symptomatic relief from edema. In some series, improvement was reported qualitatively, whereas others provided quantitative KPS changes. The overall pooled proportion was 80.7% (95% CI: 66.8%–89.6%), with moderate heterogeneity (I² = 39.9%) (Fig. 2a). In stratified analyses by tumor type, estimates for brain metastasis and mixed cohorts were broadly similar to the overall result. The HGG subgroup included one study (k = 1); therefore, the subgroup display represents a single-study estimate rather than a pooled meta-analysis, and between-group differences are described narratively. Overall, the findings support a clinically significant improvement in neurological function following resection of symptomatic RN.

RN diagnostic criteria and neurological improvement definitions varied substantially across studies (Table 2), ranging from strict 0% tumor thresholds to threshold-based

**Table 2:** Variability in radionecrosis and neurological improvement definitions

Study	RN definition category	Tumor threshold	Neurological improvement assessment
McPherson C, 2004	Mixed pathology allowed	RN as “predominant finding”	KPS, neurological deficits, steroid dose
Rusthoven K, 2011	Threshold-based	≥ 80% necrosis (< 20% tumor allowed)	Survival outcomes (symptomatic improvement not systematically assessed)
Telera S, 2013	Stratified approach	Pure RN vs. RN + recurrence	KPS, edema resolution, steroid reduction, neurological exam
Grossman R, 2016	Threshold-based	> 80% necrosis (< 20% tumor allowed)	KPS (> 70 threshold), edema resolution, steroid discontinuation
Shah A, 2019	Strict (immune-histochemical confirmation)	0% tumor (Ki-67, phosphohistone H3 negative)	KPS increase, neurological exam improvement, steroid freedom
Campos B, 2020	Strict + clinical confirmation	0% tumor + 2-year recurrence-free follow-up	Specific deficits (paresis, hemiplegia), permanent morbidity
Kim J, 2022	Stratified (3 categories)	Pure RN: 0%; Mixed: <30%; PD: >30% tumor	KPS change, specific neurological deficits (no predefined threshold)
Bhatia R, 2024	Strict	0% tumor (any tumor = progression)	Need for intervention (no systematic neurological assessment)

*KPS* Karnofsky Performance Status, *PD* progressive disease, *RN* radionecrosis. RN definitions ranged from strict 0% tumor thresholds to threshold-based approaches (< 20% tumor) to allowing RN as “predominant finding” with scattered tumor cells. Neurological improvement was defined inconsistently or not explicitly defined across studies

approaches (< 20% tumor) to less stringent criteria. This heterogeneity contributes to the moderate statistical heterogeneity (I² = 39.9%) observed.

By outcome domain (Table 3), neurological improvements were observed in approximately half of the patients, with a KPS improvement reported in 54% (moderate heterogeneity, I² = %). Radiological improvement of edema was common (75.8%) but varied considerably across studies (high heterogeneity, I² = 77.8%).

**Table 3:** Pooled incidence of different neurological outcomes following surgical resection for brain radionecrosis

Neurological Outcome	# events	Total patients assessed	Studies	Pooled incidence (95% CI)
KPS Improvement	27	50	3	54% (40.2%, 67.2%)
Motor Function Improvement	1	11	1	9.1% (1.3%, 43.9%)
Edema Resolution (Radiographic)	25	33	2	75.8% (58.5%, 87.4%)
Neurologic improvement (any domain)*	110	154	5	71.4% (63.6%, 78.1%)

*CI* confidence interval, *KPS* Karnofsky Performance Status; *Neurologic improvements include improvements in KPS, motor function, and radiographic edema resolution, which may overlap; pooled values reflect unique patients with any reported neurological improvement, including those not categorized above

### Overall survival

Reporting of overall survival after surgical resection for RN varied substantially across the included studies, both in terms of time origin (i.e., the starting point, e.g., time of radiation therapy or time of surgery, from which the duration to an event, e.g., death, is measured) and RN definition (Table 1). Three studies [25, 27, 29] reported survival from the time of resection for histopathology-confirmed pure RN. However, Shah et al. [27] included patients with benign diagnoses (meningioma, AVM) alongside malignant tumors, which could bias survival estimates. To ensure comparability, we restricted quantitative pooling to the two studies [25, 29] with exclusively malignant primary tumors (HGG and brain metastases) and report Shah et al.‘s findings descriptively.

In the pure RN cohorts, Kim et al. (2022) [29] reported a median OS of 43.7 (range 34.7–51.8) months, with 1-year and 2-year OS of 82% and 75%, respectively. Telera et al. (2013) [25] reported a median OS of 19 months (95% CI: 7–49) from resection, in a cohort that included 7 patients with pure RN and 8 with mixed RN/tumor; subgroup-specific median OS for pure RN was not reported. The pure RN subgroup demonstrated a 1-year OS of 85.7% and a 2-year OS of 68.6%. Shah et al. (2019) [27] did not report median OS but reported 1-year and 2-year OS of 93.3% and 66.7%, respectively. Although these three studies showed broadly similar short-term survival, differences in underlying populations (BM vs. mixed) and follow-up durations remained.

Because these two cohorts reported survival from a comparable time origin (resection), their 12-month and 24-month survival rates were pooled to summarize early postoperative prognosis. The pooled 12-month OS was 84.6% (95% CI: 65.4%–94.1%) (Fig. 2b), and the pooled 24-month OS was 73.1% (95% CI: 53.3%–86.6%) (Fig. 2c). In contrast, the remaining studies reported survival from non-comparable time origins, most commonly from initial diagnosis or from prior radiation therapy, and often included mixed RN and tumor-recurrence populations. Rusthoven et al. (2011) [24] reported a median survival of 21.8 months from reoperation for patients with ≥ 80% necrosis, an RN definition not restricted to pure RN, along with survival curves from diagnosis. Grossman et al. (2016) [26] reported survival exclusively from the time of diagnosis, with a median OS of 15 months for RN and 17.5 months for tumor recurrence. This unexpected result, although not statistically significant, is interpreted by the authors as secondary to selection bias. Bhatia et al. (2024) [30] reported OS primarily from the first or second SRS, again not from surgery. Other studies [23, 28] either lacked OS estimates or did not specify the time origin clearly.

Given the heterogeneity of time zero, variation in RN definitions, and differences in underlying tumor biology, a pooled quantitative OS estimate was not calculated. Instead, OS findings are presented descriptively to accurately reflect the available evidence without introducing methodological bias.

### Surgical complications

Across individual series, overall complication rates ranged from 1% to 35%, with an overall pooled estimate of 21.4% (95% CI 11.7–35.9%; I² = 61.1%); this estimate should be interpreted in the context of the reported heterogeneity and a small sample size (Fig. 2d). In stratified analyses by tumor type, heterogeneity was highest in BM cohorts **(**I² = 77.4%**)**, moderate in mixed cohorts **(**I² = 43.2%), and absent in HGG **(**I² = NA%**)**. The HGG subgroup comprised a single study (k = 1). No perioperative deaths were reported.

Postoperative complications that were observed in patients were most frequently motor deficits (5.7%) and visuospatial deficits (7.5%) (Table 4). Infections such as meningitis were also reported in some series, though less frequently. Complications were reported descriptively in the included studies, and no standardized severity grading system was applied (Appendix 2). Because patients may experience improvements in multiple neurological areas while also developing complications, these estimates can overlap.

**Table 4:** Pooled incidence of postoperative complications following surgical resection for brain radionecrosis

Complication	Events	Total patients assessed	Studies	Pooled incidence (95% CI)
Motor deficit	10	175	6	5.7 (3.1%, 10.3%)
Speech and Language	3	29	2	10.3 (3.4%, 27.6%)
Visuospatial deficit	4	53	3	7.5 (2.9%, 18.4%)
Cognitive impairment	3	45	2	6.7 (2.2%, 18.7%)
Seizures	3	53	3	5.7 (1.8%, 16.1%)
Overall complications*	20	189	7	10.6 (6.96%, 15.78%)

*CI* confidence interval; k = number of studies contributing to the outcome

*Complications may overlap across categories; pooled values reflect unique patients with any reported postoperative complication

### Quality assessment and risk of bias

Using the JBI checklist for case series, we assessed the quality of the included studies as moderate to good (Appendix 3). Among the eight studies, seven were rated as having good quality (≥ 80% of checklist criteria met), while one was rated as having moderate quality. In addition to outlining patient demographics, therapies, and results, all studies presented clear inclusion criteria and objectives. A common bias was the non-random selection of patients for surgery instead of conservative care, which may have influenced outcomes; for instance, patients with more severe symptoms were more likely to undergo resection. Comparisons across the subgroups of tumor types were also affected by external factors, as none of the studies included a control group. While most studies provided adequate clinical assessments and follow-up imaging, some outcomes, such as “neurological improvement,” were assessed subjectively and defined inconsistently. Nevertheless, all studies reported adequate follow-up durations for capturing outcomes, and exposures and outcomes were verified through imaging or histopathology. No study was deemed to carry serious bias, and none were excluded based on quality. However, as retrospective case series, these studies cannot be considered the basis for strong evidence and are of limited generalizability. Therefore, findings should be interpreted with caution, and prospective comparative studies are needed to confirm the relative efficacy of surgical versus conservative management.

## Discussion

In this study we conducted a meta-analysis to evaluate the clinical outcomes of surgical resection for symptomatic brain RN, focusing on neurological improvement, overall survival, and postoperative complications. Three key findings emerged. First, surgical resection was associated with neurological improvement in the majority of patients. These clinical benefits included enhanced performance status, motor recovery, and resolution of perilesional edema. The findings suggest that surgery may offer meaningful symptom relief in patients with a symptomatic mass effect. Second, in the subset of cohorts with pure RN and survival measured from resection, overall survival was higher at one year but declined by two years, with brain-metastasis cohorts appearing to fare better than HGG cohorts, although this difference may partly reflect reporting bias, small sample sizes, and heterogeneity in follow-up. Third, the overall postoperative complication rate was moderate, with most adverse events being manageable and non-life-threatening. These findings may inform clinical decision-making and support individualized management of refractory RN.

Survival outcomes following resection of RN remain difficult to compare across studies because of inconsistent RN definitions, variation in underlying tumor biology, and non-uniform survival time origins. Only two studies reported survival from the time of surgical resection in pure RN cohorts, which is the most clinically relevant estimand, and these were used to derive pooled 12- and 24-month survival estimates. One study [27] was excluded from pooling due to the inclusion of benign diagnoses. Although these studies suggested favorable 1-year survival (on the order of 80%–90%) with a decline by 2 years, the estimates should be interpreted cautiously given the small sample sizes, heterogeneity in pathology (BM vs. HGG), and variability in follow-up.

By contrast, several other studies reported overall survival from diagnosis or from prior radiotherapy rather than from surgery, reflecting a different prognostic trajectory driven by primary tumor behavior, initial treatment, and systemic therapies. Including such heterogeneous survival definitions in a single pooled analysis would artificially inflate or distort expectations after RN surgery. We therefore restricted quantitative pooling of overall survival to the two pure RN cohorts with a common, surgery-based time origin and presented the remaining survival findings descriptively. This approach is consistent with methodological standards for time-to-event outcomes and preserves clinical interpretability while avoiding misleading summary estimates [31, 32].

The available literature is concordant with the beneficial role of surgical treatment for cerebral RN on symptom relief and neurological status, in appropriately selected patients [33, 34]. For instance, a prior systematic review reported that surgical resection for symptomatic RN is associated with symptom improvement or stability in approximately 89% of patients, with reported severe complication rates ranging from 0% to 2% and overall survival ranging from 19 to 86 months across small retrospective series [7]. Mechanisms that have been proposed for neurological symptom improvement are alleviation of mass effect, reduction of peri-lesional edema, and interruption of the inflammatory cascade [33]. An important clinical benefit of surgery is the reduction or discontinuation of corticosteroids. Radiographic edema resolution occurred in 75.8% of cases, typically enabling steroid tapering or elimination. Given the substantial morbidity of chronic corticosteroid therapy, including immunosuppression, myopathy, psychiatric effects, and metabolic derangement [35, 36], this represents a major therapeutic advantage. For patients requiring high-dose steroids or those developing steroid-related complications, surgery may offer the only viable path to steroid independence. A critical limitation concerns heterogeneity in timing from radiation to RN development. While RN classically manifests as a late effect occurring months to years after treatment, some included series reported individual cases as early as 1 month [24] or 2 months [30] post-radiation. Such early-onset presentations more likely represent acute radiation injury or pseudoprogression rather than the chronic vascular injury characteristic of late RN. This biological heterogeneity may confound outcome interpretation, as early inflammatory processes could respond differently to surgical intervention than established chronic necrosis.

While our findings support surgical resection for symptomatic RN, the current evidence does not permit definitive patient selection criteria. In clinical practice, suspected RN is initially managed with diagnostic evaluation and corticosteroid therapy, with consideration of bevacizumab in steroid-refractory cases. Based on patterns observed across included studies, surgery was most commonly performed in patients with progressive, symptomatic lesions refractory to medical management (particularly corticosteroids) with significant mass effect. The observed neurological improvement rate of 80.7% and potential for steroid reduction suggest surgery may be valuable in such cases [33, 34]. However, decisions must remain individualized through multidisciplinary discussion, weighing lesion location, patient functional status, surgical risks, and patient preferences. Standardized reporting of surgical indications and survival from the time of resection will be necessary to establish evidence-based selection criteria.

The interpretation of our findings is influenced by several limitations. First, this meta-analysis is limited by its retrospective design and the absence of comparator groups. All included studies were observational case series without concurrent controls receiving medical therapy (e.g., bevacizumab) or alternative interventions (e.g., laser interstitial thermal therapy). Therefore, causal inference is not possible, and observed improvements cannot be definitively attributed to surgery. Furthermore, surgical cohorts likely represent more severe or refractory cases, introducing potential selection bias. These limitations suggest that while surgery may provide clinical benefit, its superiority over conservative management cannot be established.

Second, clinical and methodological heterogeneity limited the robustness and generalizability of our analyses. Of 443 patients, only 141 (32%) had histopathologically confirmed pure radionecrosis, and there was substantial variation in tumor type (high-grade glioma, metastases, benign diagnoses like meningioma), radiation modality (stereotactic radiosurgery vs. fractionated radiotherapy), and timing of radionecrosis (from 1 month to years post-radiation). Key clinical factors, including systemic therapies (chemotherapy, immunotherapy), re-irradiation, and baseline functional status, were not consistently controlled. Most studies did not stratify outcomes by lesion volume, anatomical location (supratentorial vs. infratentorial, eloquent vs. non-eloquent cortex), or lesion depth, all of which are known to affect surgical risk and postoperative outcomes. Diagnostic criteria for radionecrosis and definitions of neurological improvement were also inconsistent. Survival data were heterogeneous, with only two studies providing comparable post-resection survival outcomes.

Third, critical gaps in procedural and clinical reporting prevented comprehensive analysis of surgical decision-making and technique-outcome relationships. Studies did not systematically report surgical indications, the timing of intervention relative to symptom onset, or baseline symptom profiles, precluding identification of factors predicting surgical benefit. Similarly, surgical procedural details, including extent of resection (gross total versus subtotal), surgical approach (standard craniotomy versus decompressive craniectomy), and use of adjunctive measures such as expansile duraplasty, were inconsistently documented, preventing analysis of how technical factors influence outcomes. Future investigations should prioritize standardized reporting of these variables alongside larger, well-characterized cohorts with uniform survival measurement from the time of resection to strengthen the evidence base.

These limitations warrant cautious interpretation of our findings. Prospective controlled comparative studies with standardized outcome reporting are needed to better define the role of surgical resection in symptomatic brain radionecrosis.

This study has significant strengths despite its limitations. To our knowledge, this report is the first quantitative meta-analysis to examine the association between surgical resection and neurological outcomes and survival in patients with brain RN. It enhances the body of evidence as it represents one of the most extensive series of surgically resected cases of histologically confirmed cerebral RN. Focusing on therapeutic relevance, we evaluated neurological improvement as our primary endpoint. Because few studies statistically describe neurological recovery, this emphasis on functional outcomes rather than just radiographic improvements addresses a gap in the research and aligns with patient-centered goals. Finally, another strength of our review was that when survival data were only available in graphical form, we extracted these values using validated digitization software (WebPlotDigitizer), ensuring that time-to-event outcomes could still be included in the pooled analysis. These advantages enhance our findings and the applicability of our recommendations in clinical settings.

Our findings underscore significant gaps that hinder evidence-based decision-making. Future studies should emphasize the standardized reporting of patient characteristics, including primary tumor type, radiation parameters, and histopathological confirmation of RN status. It is also important to assess preoperative neurological status using validated scales such as KPS, ECOG, and mRS, and to document surgical variables such as indication criteria, extent of resection, and lesion characteristics. Outcomes should be evaluated at consistent intervals using standardized measures that encompass domain-specific neurological function, steroid dosage trajectories, and survival rates, all with clearly defined time origins. Complications must be graded using recognized severity systems. Most importantly, prospective controlled studies comparing surgical resection with medical therapies (e.g., bevacizumab) or alternative interventions (e.g., laser interstitial thermal therapy) are imperative. Establishing multi-institutional registries would facilitate adequate sample sizes for subgroup analyses and aid in identifying predictors of surgical benefit, ultimately bolstering the evidence base for patient selection.

## Conclusion

Surgical resection of symptomatic brain RN was associated with favorable neurological outcomes and an acceptable safety profile. Descriptive survival data suggested higher overall survival at one year, with a decline by two years in the available pure RN cohorts. Resection may be considered a valuable option to improve neurological outcomes and quality of life. Future well-designed, comparative, and longitudinal studies should identify predictors of surgical benefit and include more rigorous comparisons between resection and conservative management.

## Supplementary Information

Below is the link to the electronic supplementary material.

Supplementary Material 1

## Data Availability

No datasets were generated or analysed during the current study.
