# Supplementary material for: Resection of brain radionecrosis after stereotactic radiosurgery or radiotherapy: a meta-analysis
Source: Neurosurg Rev. 2026 Mar 14;49(1):279. doi: 10.1007/s10143-026-04208-x (PMC12987881; doi:10.1007/s10143-026-04208-x)
Supplement: Supplementary file 1 — Supplementary Material 1 [file 10143_2026_4208_MOESM1_ESM.docx]

**Resection of brain radionecrosis after stereotactic radiosurgery or radiotherapy: A meta-analysis.**

Karun Donthineni^1^, Hyejoong M. Lee^1^, Prabodh Sankhe^1^, Alice van den Broek^2^, Semah Misconi^2^, Charissa Jessurun^2,3^, Marco Mammi^4^, Marike L.D Broekman*^2^, Rania A. Mekary*^1,5,6^

1. School of Pharmacy, MCPHS University, Boston, MA 02215, USA
2. Department of Neurosurgery, Haaglanden Medical Center and Leiden University Medical Center, Leiden, The Netherlands
3. Dept of Radiotherapy, Amsterdam University Medical Center, Amsterdam, The Netherlands
4. Department of Neurosurgery, “M. Bufalini” Hospital, Cesena, Italy
5. Department of Neurosurgery, Brigham and Women's Hospital, Boston, MA, USA.
6. Department of Biostatistics, Harvard TH Chan School of Public Health, Boston, MA, USA.

* co-senior authors

**Appendices**

**Appendix 1:** Search Strategy (02/28/25)

| **S.no** | **PubMed Search Terms** | **Hits** |
| --- | --- | --- |
| 1 | (Necrosis [Mesh] OR radionecrosis [tw] OR radiation necrosis [tw] OR post-radiation necrosis [tw] OR radiation-induced injury [tw])  AND  (Brain [Tw] OR Brain [MeSH Terms] OR Cerebrum [MeSH Terms] OR Intracranial [Tw] OR Cerebr*[Tw] OR Cerebell*[Tw]) | 61,603 |
| 2 | (Radiosurgery[Mesh] OR Radiotherapy[Mesh] OR Dose fractionation [MeSH] OR Cranial Irradiation [MeSH] OR stereotactic radiosurgery[tw] OR SRS[tw] OR stereotactic radiotherapy[tw] OR SRT[tw] OR WBRT [tw] OR SFRT [tw] OR brain radiosurgery[tw] OR radio surgical procedures[tw] OR precision radiotherapy[tw] OR fractionated radiotherapy[tw] OR gamma knife[tw] OR cyberknife[tw] OR radiation therapy[tw] OR LINAC [tw] OR linear accelerator [tw] OR x knife[tw] OR stereotactic*[tw]) | 306,561 |
| 3 | (Craniotomy [Mesh] OR Neurosurgical Procedures [Mesh:NoExp] OR Neurosurgery [Mesh] OR Brain/Surgery [Mesh] OR Surgical Procedures, Operative[MeSH Terms] OR neurosurg* [tw] or brain surg*[tw] OR resect*[Tw]) | 3,885,706 |
| 4 | #1 AND #2 AND #3 | **1,225** |

| **S.no** | **Embase Search Terms** | **Hits** |
| --- | --- | --- |
| 1 | (Necrosis/ OR Brain/ OR "radionecrosis" or "radiation necrosis" or "post-radiation necrosis" or "radiation-induced injury").tw. AND (Brain/ OR Cerebrum/ OR "Brain" OR "Intracranial" OR Cerebr* OR Cerebell*).tw. | 8,926 |
| 2 | (Radiosurgery/ OR Radiotherapy/ OR Dose fractionation/ OR Cranial irradiation/ OR "stereotactic radiosurgery" OR "SRS" OR "stereotactic radiotherapy" OR "SRT" OR WBRT OR SFRT OR "brain radiosurgery" OR "radio surgical procedures" OR "precision radiotherapy" OR "fractionated radiotherapy" OR "gamma knife" OR "cyberknife" OR "radiation therapy" OR "LINAC" OR "linear accelerator" OR "x knife" OR stereotactic*).tw. | 370,628 |
| 3 | (Craniotomy/ OR Neurosurgery/ OR Neurosurgical Procedures/ OR Surgical Procedures Operative/ OR neurosurg* or brain surg*).tw. | 901,923 |
| 4 | #1 AND #2 AND #3 | **772** |

| S.no | **Cochrane Search terms** | Hits |
| --- | --- | --- |
| 1 | ("craniotomy" OR "neurosurgical procedures" OR "neurosurgery" OR "Brain/surgery" OR cranitom* OR neurosurg* OR brain NEXT surg* OR Surg* OR resect* OR operation* OR operativ*):ti,ab,kw | 366,145 |
| 2 | ("stereotactic radiosurgery" OR "SRS" OR "stereotactic radiotherapy" OR "SRT" OR WBRT OR SFRT OR "brain radiosurgery" OR "radio surgical procedures" OR "precision radiotherapy" OR "fractionated radiotherapy" OR "gamma knife" OR "cyberknife" OR "radiation therapy" OR Radiotherap* OR Radiosurg* OR LINAC OR linear NEXT accelerator OR x knife OR stereotactic* OR fraction* OR irradiat* OR radiat*):ti,ab,kw | 101,701 |
| 3 | ("radionecrosis" OR "brain necrosis" OR "cerebral radionecrosis" OR "radiation necrosis" OR "cerebral necrosis" OR "post-radiation necrosis" OR "radiation induced brain injury" OR "radiation-induced cerebral injury") AND (Brain OR Cerebr*OR Cerebell* OR Intracranial):ti,ab,kw | 9,990 |
| 4 | MeSH descriptor: [Neurosurgery] explode all trees | 146 |
| 5 | MeSH descriptor: [Necrosis] explode all trees | 18,748 |
| 6 | MeSH descriptor: [Dose Fractionation, Radiation] explode all trees | 1,130 |
| 7 | MeSH descriptor: [Radiotherapy] explode all trees | 9,984 |
| 8 | MeSH descriptor: [Craniotomy] explode all trees | 663 |
| 9 | #1 OR #4 OR #8 | 366,152 |
| 10 | #2 OR #6 OR #7 | 102,023 |
| 11 | #3 OR #5 | 28,220 |
| 12 | #5 AND #6 AND #7 | **4** |

**Appendix 2: Individual outcomes reported in each study**

| **Reference** | **Neurological Outcome** | **Post-operative complication** |
| --- | --- | --- |
| McPherson C, 2004 | N: 4/11  - KPS improved: 4;  - Hemiparesis improved: 1 | N: 3/11  - worse Hemiparesis: 1(pt 5);  - worsening of Gait: 1(pt 6);  - new visual field deficit: 2 (pt 4,6) |
| Rusthoven KE, 2011 | NR | N: 1/14  - progressive speech dysfunction: 1;  - recurrent focal seizures: 1 |
| Telera S, 2013 | N: 14/15  - KPS improved: 7;  - Brain edema progressively resolved in all cases  - 14 patients, except for one (which presented a severe deficit to the right arm), remained neurologically stable or improved. | N: 4/15  - transient cerebellar ataxia: 1;  - transient dysphasia: 2;  - post-operative seizure: 1 |
| Grossman R, 2016 | N: 10/18  - decrease in the extent of brain edema: 10 | N: 6/18  - Post-operative complication: 6 (35.3%), p-value: 0.1;  - New motor deficit:  2/6 (33.3%);  - New visual deficit: 1/6 (16.7%) |
| Shah AH, 2019 | N: 16/24  - Most patients (66.7%) in our series experienced postoperative KPS improvement: 16;  - Neurological improvement at latest exam: 16;  - Latest follow-up: 72.3% demonstrated neurological improvement. | N: 6/24  - L-sided weakness, L-leg weakness, inability to move: 3;  - L homonymous hemianopsia: 1;  - difficulty concentrating, Mental status changes: 2;  - focal seizures: 1 |
| Campos B, 2020 | NR | N; 2/21  - Aggravated paresis: 1; |
| Kim J, 2022 | N: 66/86  - Neurological deficits improved for most patients (66, 77%) | N: 1/86  - Hemiplegia: 1 |
|  | Total: 110/ 154 (N/Total) * | Total: 20 / 189 (N/Total) ** |

Individual patient outcomes are reported as mentioned in the original studies. Outcomes listed include improvements and complications observed after surgical intervention for brain radionecrosis.

Some outcome descriptions were quoted directly from the original articles due to lack of quantifiable data.

**Abbreviations:** KPS: Karnofsky Performance Status; NR: not reported; pt: patient; L: left

*Improvements in KPS, motor function, and resolution of perilesional edema may overlap; total N reflects unique patients with any neurological improvement.

**Postoperative complications may also overlap across categories; total N reflects unique patients who experienced any complication.

**Appendix 3**: Joanna Briggs Institute (JBI) checklist for Case series

| **Quality assessment performed using the questionnaire by JBI checklist** | | | | | | | | |
| --- | --- | --- | --- | --- | --- | --- | --- | --- |
|  | **McPherson C, 2004** | **Rusthoven K.E., 2011** | **Telera S, 2013** | **Grossman R, 2016** | **Shah A, 2019** | **Campos B, 2020** | **Kim J, 2022** | **Bhatia R, 2024** |
| **Was there clear criteria for inclusion in the case series?** | Yes | Yes | Yes | Yes | Yes | Yes | Yes | Yes |
| **Was the condition measured in a standard, reliable way for all participants included in the case series?** | Yes | Yes | Yes | Yes | Yes | Yes | Yes | Yes |
| **Were valid methods used for identification of the condition for all participants included in the case series ?** | Yes | Yes | Yes | Yes | Yes | Yes | Yes | Yes |
| **Did the case series have consecutive inclusion of participants ?** | Yes | Unclear | Yes | Yes | Yes | Yes | Yes | Unclear |
| **Did the case series have complete inclusion of participants?** | Yes | Unclear | Yes | Yes | Yes | Yes | Yes | Unclear |
| **Was there clear reporting of the demographics of the participants in the study?** | Yes | Yes | Yes | Yes | Yes | Yes | Yes | Yes |
| **Was there clear reporting of clinical information of the participants ?** | Yes | Yes | Yes | Yes | Yes | Yes | Yes | Yes |
| **Were the outcomes or follow-up results of cases clearly reported?** | Yes | Yes | Yes | Yes | Yes | Yes | Yes | Yes |
| **Was there clear reporting of the presenting sites/clinics' demographic information?** | Yes | No | Yes | Yes | Yes | Yes | Yes | Yes |
| **Was statistical analysis appropriate?** | No | Yes | Yes | Yes | Yes | Yes | Yes | Yes |
| **Final Quality Score (%)** | 90 | 70 | 100 | 100 | 100 | 100 | 100 | 80 |

Final quality scores calculated as percentage of 'Yes' responses out of total items (10) on the Joanna Briggs Institute (JBI) Critical Appraisal Checklist for Case Series.
